# Tricyclo­[3.3.1.0^3,7^]nonane-3,7-diyl bis­(methane­sulfonate)

**DOI:** 10.1107/S1600536810001261

**Published:** 2010-01-20

**Authors:** Savvas Ioannou, Athanassios V. Nicolaides, Manolis J. Manos

**Affiliations:** a Department of Chemistry, University of Cyprus, 1678 Nicosia, Cyprus

## Abstract

The crystal structure of the title compound, C_11_H_18_O_6_S_2_, was determined to investigate the effect of the eclipsed mesyl groups on the bond length of the vicinal quaternary C atoms. The two quaternary C atoms of the noradamantane skeleton and the two O atoms to which they are connected all located essentially in the same plane [maximum deviation 0.01 Å], resulting in an eclipsing conformation of the C—O bonds. The C—C bond of the quaternary C atoms is 1.597 (3) Å is considerably longer than the other C—C bonds of the mol­ecule.

## Related literature

For reviews on noradamantene and analogous pyramidalized alkenes, see: Borden (1989 ▶, 1996 ▶); Vázquez & Camps (2005 ▶). For the syntheses of mesylate esters of acyclic alcohols, see: Danheiser *et al.* (1988 ▶); Marshall & Chobanian (2005 ▶). For the synthesis of the precursor diol (tricyclo-[3.3.1.0^3,7^]nonane-3,7-diol), an important inter­mediate in the synthetic route towards the generation of noradamantene, see: Zalikowski *et al.* (1980 ▶); Bertz (1985 ▶). For the synthesis of the title compound, see: Ioannou & Nicolaides (2009 ▶).
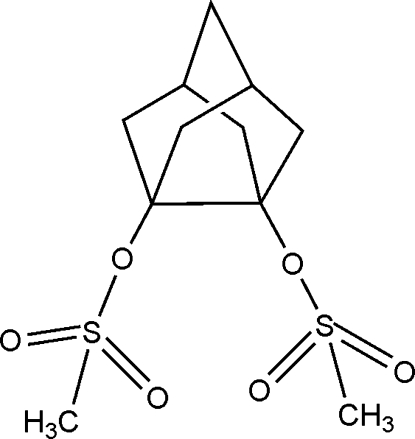


## Experimental

### 

#### Crystal data

C_11_H_18_O_6_S_2_
*M* *_r_* = 310.37
Monoclinic, 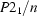

*a* = 8.8017 (2) Å
*b* = 10.3107 (2) Å
*c* = 14.4623 (3) Å
β = 92.092 (2)°
*V* = 1311.60 (5) Å^3^
*Z* = 4
Mo *K*α radiation
μ = 0.43 mm^−1^
*T* = 100 K
0.18 × 0.06 × 0.04 mm

#### Data collection

Oxford Diffraction Xcalibur-3 diffractometer
Absorption correction: multi-scan (*CrysAlis RED*; Oxford Diffraction, 2008 ▶) *T*_min_ = 0.919, *T*_max_ = 1.000
8431 measured reflections
2308 independent reflections
1791 reflections with *I* > 2σ(*I*)
*R*_int_ = 0.032

#### Refinement

*R*[*F* ^2^ > 2σ(*F* ^2^)] = 0.029
*wR*(*F* ^2^) = 0.073
*S* = 1.00
2308 reflections
172 parameters
H-atom parameters constrained
Δρ_max_ = 0.31 e Å^−3^
Δρ_min_ = −0.34 e Å^−3^

### 

Data collection: *CrysAlis CCD* (Oxford Diffraction, 2008 ▶); cell refinement: *CrysAlis RED* (Oxford Diffraction, 2008 ▶); data reduction: *CrysAlis RED*; program(s) used to solve structure: *SHELXS97* (Sheldrick, 2008 ▶); program(s) used to refine structure: *SHELXL97* (Sheldrick, 2008 ▶); molecular graphics: *WinGX* (Farrugia, 1999 ▶); software used to prepare material for publication: *WinGX*.

## Supplementary Material

Crystal structure: contains datablocks I, global. DOI: 10.1107/S1600536810001261/nc2172sup1.cif

Structure factors: contains datablocks I. DOI: 10.1107/S1600536810001261/nc2172Isup2.hkl

Additional supplementary materials:  crystallographic information; 3D view; checkCIF report

## Figures and Tables

**Fig. 1.**
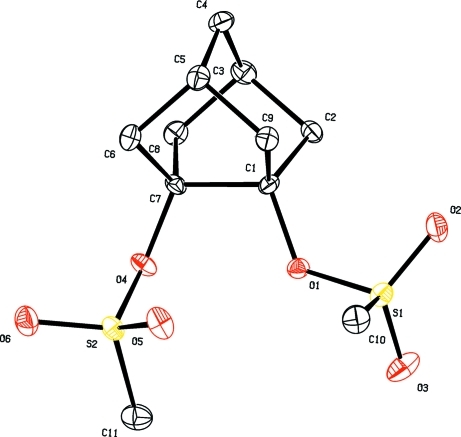
Structure of the title compound tricyclo-[3.3.1.03,7]nonane-3,7-diyldimesylate with the atom-labelling scheme. Displacement ellipsoids are drawn at the 50% probability level. Hydrogen atoms have been omitted for clarity.

## supplementary crystallographic information

### Experimental

Synthesis of tricyclo-[3.3.1.0^3,7^]nonane-3,7-diyl dimesylate (1). To a solution of tricyclo-[3.3.1.0^3,7^]nonane-3,7-diol (1.00 g, 6.49 mmol) in pyridine (10 ml), mesyl chloride (CH_3_SO_2_Cl)(5.02 ml, 65 mmol) was added slowly with stirring at ambient temperature. The mixture was then heated at 120 *^o^*C for 5 h. After cooling, crushed ice (100 g) was added and the mixture extracted with CH_2_Cl_2_ (5 *x* 20 ml). The combined organic phase was washed with 2*M* HCl (2 *x* 40 ml), H_2_O (2 *x* 20 ml), saturated aqueous NaHCO_3_ (2 *x* 20 ml), and dried (Na_2_SO_4_). After filtration and removal of the solvent under reduced pressure, a brown solid (1.92 g, 96%) was isolated. Recrystallization from THF/hexane afforded pure 1 (1.71 g, 85%) as colorless crystals m.p. 127–128 *^o^*C. Elemental analysis (%): Calculated for C_11_H_18_O_6_S_2_: *C*,42.6; H, 5.8; O, 30.9; S, 20.7. Found: C, 42.3; H, 5.7; S,20.3. High-resolution Mass Spectrometry (TOF MS ES+): Calculated for C_11_H_19_O_6_S_2_ 311.0623 found: 311.0629. ν_max_(KBr) 3449, 2943, 1464, 1414, 1341, 1190, 1169, 1101, 1018, 976, 955, 856, 824, 802, 760, 669, 615, 565, 515, 474 cm^-1^; *δ*H(300 MHz, CDCl_3_) 3.10 (6*H*, –CH_3_, s), 2.50 (6H(4eq+2CH), d, J 6.9 Hz), 2.26 (4Hax,d, J 9.0 Hz), 1.51 (2Hbridge, s); *δ*^13^C (75.5 MHz, CDCl_3_) 91.30 (–CO), 47.42(–CH_2_),40.60 (–CH_3_), 34.98 (–CH), 32.28 (–CH_2_ bridge).

### Refinement

The H atoms were positioned with idealized geometry and refined using a riding model with *U*_iso_(H) = 1.2 or 1.5 (methyl H atoms) of *U*_eq_(C).

### Crystal data

**Table:**

C_11_H_18_O_6_S_2_	*F*(000) = 656
*M**_r_* = 310.37	*D*_x_ = 1.572 Mg m^−^^3^
Monoclinic, *P*2_1_/*n*	Mo *K*α radiation, λ = 0.71073 Å
Hall symbol: -P 2yn	Cell parameters from 4520 reflections
*a* = 8.8017 (2) Å	θ = 3.0–30.2°
*b* = 10.3107 (2) Å	µ = 0.43 mm^−^^1^
*c* = 14.4623 (3) Å	*T* = 100 K
β = 92.092 (2)°	Plate, colorless
*V* = 1311.60 (5) Å^3^	0.18 × 0.06 × 0.04 mm
*Z* = 4	

### Data collection

**Table:**

Oxford Diffraction Xcalibur-3 diffractometer	2308 independent reflections
Radiation source: fine-focus sealed tube	1791 reflections with *I* > 2σ(*I*)
graphite	*R*_int_ = 0.032
Detector resolution: 16.0288 pixels mm^-1^	θ_max_ = 25.0°, θ_min_ = 3.0°
ω scans	*h* = −6→10
Absorption correction: multi-scan (*CrysAlis RED*; Oxford Diffraction, 2008)	*k* = −12→12
*T*_min_ = 0.919, *T*_max_ = 1.000	*l* = −17→17
8431 measured reflections	

### Refinement

**Table:**

Refinement on *F*^2^	Primary atom site location: structure-invariant direct methods
Least-squares matrix: full	Secondary atom site location: difference Fourier map
*R*[*F*^2^ > 2σ(*F*^2^)] = 0.029	Hydrogen site location: inferred from neighbouring sites
*wR*(*F*^2^) = 0.073	H-atom parameters constrained
*S* = 1.00	*w* = 1/[σ^2^(*F*_o_^2^) + (0.0414*P*)^2^] where *P* = (*F*_o_^2^ + 2*F*_c_^2^)/3
2308 reflections	(Δ/σ)_max_ = 0.001
172 parameters	Δρ_max_ = 0.31 e Å^−^^3^
0 restraints	Δρ_min_ = −0.34 e Å^−^^3^

### Special details

**Table:**

Geometry. All e.s.d.'s (except the e.s.d. in the dihedral angle between two l.s. planes) are estimated using the full covariance matrix. The cell e.s.d.'s are taken into account individually in the estimation of e.s.d.'s in distances, angles and torsion angles; correlations between e.s.d.'s in cell parameters are only used when they are defined by crystal symmetry. An approximate (isotropic) treatment of cell e.s.d.'s is used for estimating e.s.d.'s involving l.s. planes.
Refinement. Refinement of *F*^2^ against ALL reflections. The weighted *R*-factor *wR* and goodness of fit *S* are based on *F*^2^, conventional *R*-factors *R* are based on *F*, with *F* set to zero for negative *F*^2^. The threshold expression of *F*^2^ > σ(*F*^2^) is used only for calculating *R*-factors(gt) *etc*. and is not relevant to the choice of reflections for refinement. *R*-factors based on *F*^2^ are statistically about twice as large as those based on *F*, and *R*- factors based on ALL data will be even larger.

### Fractional atomic coordinates and isotropic or equivalent isotropic displacement parameters (Å^2^)

**Table:**

	*x*	*y*	*z*	*U*_iso_*/*U*_eq_	
S1	0.15096 (6)	0.16605 (5)	−0.14000 (3)	0.01491 (14)	
S2	0.58045 (5)	0.26342 (5)	0.01791 (4)	0.01445 (14)	
O1	0.24294 (14)	0.17383 (12)	−0.04474 (9)	0.0127 (3)	
O2	−0.00837 (15)	0.17596 (14)	−0.12494 (10)	0.0203 (4)	
O3	0.20781 (16)	0.05237 (15)	−0.18235 (10)	0.0263 (4)	
O4	0.46545 (14)	0.19451 (13)	0.08427 (9)	0.0147 (3)	
O5	0.50094 (15)	0.33931 (14)	−0.05052 (10)	0.0204 (3)	
O6	0.69589 (15)	0.32558 (14)	0.07372 (10)	0.0213 (4)	
C1	0.1867 (2)	0.23973 (19)	0.03658 (13)	0.0118 (4)	
C2	0.0669 (2)	0.16160 (19)	0.08527 (13)	0.0141 (5)	
H2A	0.0827	0.0690	0.0784	0.017*	
H2B	−0.0351	0.1838	0.0629	0.017*	
C3	0.0968 (2)	0.2058 (2)	0.18502 (14)	0.0164 (5)	
H3	0.0386	0.1553	0.2289	0.020*	
C4	0.0647 (2)	0.3523 (2)	0.19196 (14)	0.0179 (5)	
H4A	0.0896	0.3818	0.2544	0.021*	
H4B	−0.0427	0.3681	0.1792	0.021*	
C5	0.1586 (2)	0.4297 (2)	0.12291 (14)	0.0157 (5)	
H5	0.1397	0.5231	0.1271	0.019*	
C6	0.3291 (2)	0.39729 (18)	0.13632 (15)	0.0154 (5)	
H6A	0.3908	0.4462	0.0944	0.018*	
H6B	0.3657	0.4116	0.1996	0.018*	
C7	0.3234 (2)	0.25284 (19)	0.11167 (14)	0.0122 (4)	
C8	0.2677 (2)	0.17855 (19)	0.19476 (14)	0.0150 (4)	
H8A	0.3113	0.2123	0.2524	0.018*	
H8B	0.2896	0.0866	0.1904	0.018*	
C9	0.1300 (2)	0.37854 (18)	0.02389 (14)	0.0144 (4)	
H9A	0.0231	0.3814	0.0053	0.017*	
H9B	0.1886	0.4258	−0.0206	0.017*	
C10	0.2097 (2)	0.3026 (2)	−0.20119 (15)	0.0201 (5)	
H10A	0.1715	0.3795	−0.1728	0.030*	
H10B	0.1711	0.2975	−0.2640	0.030*	
H10C	0.3188	0.3057	−0.2003	0.030*	
C11	0.6535 (2)	0.1234 (2)	−0.03142 (15)	0.0215 (5)	
H11A	0.7066	0.0734	0.0155	0.032*	
H11B	0.7223	0.1469	−0.0786	0.032*	
H11C	0.5716	0.0728	−0.0583	0.032*	

### Atomic displacement parameters (Å^2^)

**Table:**

	*U*^11^	*U*^22^	*U*^33^	*U*^12^	*U*^13^	*U*^23^
S1	0.0177 (3)	0.0144 (3)	0.0126 (3)	−0.0015 (2)	0.0004 (2)	−0.0005 (2)
S2	0.0119 (2)	0.0140 (3)	0.0175 (3)	−0.0014 (2)	0.0012 (2)	0.0022 (2)
O1	0.0132 (7)	0.0129 (7)	0.0119 (7)	0.0017 (6)	0.0005 (6)	−0.0006 (6)
O2	0.0142 (7)	0.0285 (9)	0.0179 (8)	−0.0048 (6)	−0.0022 (6)	0.0026 (7)
O3	0.0400 (9)	0.0201 (9)	0.0186 (9)	0.0031 (7)	0.0002 (7)	−0.0068 (7)
O4	0.0105 (7)	0.0148 (8)	0.0189 (8)	0.0027 (5)	0.0025 (6)	0.0048 (6)
O5	0.0169 (7)	0.0199 (8)	0.0242 (8)	−0.0022 (6)	−0.0016 (6)	0.0095 (7)
O6	0.0167 (7)	0.0224 (8)	0.0247 (9)	−0.0059 (6)	−0.0014 (6)	−0.0005 (7)
C1	0.0141 (9)	0.0114 (10)	0.0098 (10)	0.0018 (8)	−0.0004 (8)	−0.0024 (8)
C2	0.0112 (10)	0.0135 (11)	0.0177 (11)	−0.0001 (8)	0.0015 (8)	−0.0008 (9)
C3	0.0149 (10)	0.0177 (11)	0.0167 (11)	−0.0009 (9)	0.0035 (9)	0.0029 (9)
C4	0.0187 (10)	0.0205 (12)	0.0144 (11)	0.0037 (9)	0.0013 (9)	−0.0051 (9)
C5	0.0182 (10)	0.0114 (11)	0.0173 (11)	0.0027 (9)	−0.0025 (9)	−0.0015 (9)
C6	0.0174 (10)	0.0136 (11)	0.0151 (11)	−0.0028 (8)	−0.0019 (8)	−0.0010 (9)
C7	0.0086 (9)	0.0127 (11)	0.0154 (11)	0.0027 (8)	0.0006 (8)	0.0000 (8)
C8	0.0182 (10)	0.0144 (11)	0.0125 (11)	−0.0003 (9)	0.0004 (8)	0.0016 (9)
C9	0.0157 (10)	0.0107 (10)	0.0165 (11)	0.0028 (8)	−0.0022 (8)	0.0010 (9)
C10	0.0234 (11)	0.0223 (12)	0.0146 (11)	−0.0026 (9)	0.0015 (9)	0.0054 (9)
C11	0.0222 (11)	0.0187 (11)	0.0242 (12)	−0.0004 (9)	0.0068 (10)	0.0008 (10)

### Geometric parameters (Å, °)

**Table:**

S1—O3	1.4219 (15)	C4—H4A	0.9700
S1—O2	1.4307 (14)	C4—H4B	0.9700
S1—O1	1.5742 (14)	C5—C9	1.538 (3)
S1—C10	1.751 (2)	C5—C6	1.542 (3)
S2—O5	1.4251 (15)	C5—H5	0.9800
S2—O6	1.4264 (14)	C6—C7	1.532 (3)
S2—O4	1.5878 (13)	C6—H6A	0.9700
S2—C11	1.744 (2)	C6—H6B	0.9700
O1—C1	1.460 (2)	C7—C8	1.521 (3)
O4—C7	1.456 (2)	C8—H8A	0.9700
C1—C2	1.520 (3)	C8—H8B	0.9700
C1—C9	1.525 (3)	C9—H9A	0.9700
C1—C7	1.597 (3)	C9—H9B	0.9700
C2—C3	1.526 (3)	C10—H10A	0.9600
C2—H2A	0.9700	C10—H10B	0.9600
C2—H2B	0.9700	C10—H10C	0.9600
C3—C8	1.532 (3)	C11—H11A	0.9600
C3—C4	1.541 (3)	C11—H11B	0.9600
C3—H3	0.9800	C11—H11C	0.9600
C4—C5	1.542 (3)		
			
O3—S1—O2	119.13 (9)	C4—C5—C6	110.43 (16)
O3—S1—O1	103.95 (8)	C9—C5—H5	111.8
O2—S1—O1	109.80 (8)	C4—C5—H5	111.8
O3—S1—C10	109.27 (10)	C6—C5—H5	111.8
O2—S1—C10	109.20 (9)	C7—C6—C5	99.07 (14)
O1—S1—C10	104.44 (9)	C7—C6—H6A	112.0
O5—S2—O6	117.89 (9)	C5—C6—H6A	112.0
O5—S2—O4	110.96 (8)	C7—C6—H6B	112.0
O6—S2—O4	108.40 (8)	C5—C6—H6B	112.0
O5—S2—C11	110.46 (10)	H6A—C6—H6B	109.6
O6—S2—C11	109.76 (10)	O4—C7—C8	108.17 (15)
O4—S2—C11	97.43 (9)	O4—C7—C6	116.36 (15)
C1—O1—S1	123.37 (11)	C8—C7—C6	108.33 (16)
C7—O4—S2	123.55 (12)	O4—C7—C1	114.40 (15)
O1—C1—C2	112.81 (15)	C8—C7—C1	103.79 (14)
O1—C1—C9	117.32 (16)	C6—C7—C1	104.95 (15)
C2—C1—C9	108.87 (15)	C7—C8—C3	100.29 (15)
O1—C1—C7	108.56 (14)	C7—C8—H8A	111.7
C2—C1—C7	104.36 (15)	C3—C8—H8A	111.7
C9—C1—C7	103.75 (15)	C7—C8—H8B	111.7
C1—C2—C3	100.43 (15)	C3—C8—H8B	111.7
C1—C2—H2A	111.7	H8A—C8—H8B	109.5
C3—C2—H2A	111.7	C1—C9—C5	99.66 (15)
C1—C2—H2B	111.7	C1—C9—H9A	111.8
C3—C2—H2B	111.7	C5—C9—H9A	111.8
H2A—C2—H2B	109.5	C1—C9—H9B	111.8
C2—C3—C8	99.61 (15)	C5—C9—H9B	111.8
C2—C3—C4	109.18 (17)	H9A—C9—H9B	109.6
C8—C3—C4	110.84 (16)	S1—C10—H10A	109.5
C2—C3—H3	112.2	S1—C10—H10B	109.5
C8—C3—H3	112.2	H10A—C10—H10B	109.5
C4—C3—H3	112.2	S1—C10—H10C	109.5
C3—C4—C5	111.15 (16)	H10A—C10—H10C	109.5
C3—C4—H4A	109.4	H10B—C10—H10C	109.5
C5—C4—H4A	109.4	S2—C11—H11A	109.5
C3—C4—H4B	109.4	S2—C11—H11B	109.5
C5—C4—H4B	109.4	H11A—C11—H11B	109.5
H4A—C4—H4B	108.0	S2—C11—H11C	109.5
C9—C5—C4	110.63 (16)	H11A—C11—H11C	109.5
C9—C5—C6	99.70 (16)	H11B—C11—H11C	109.5
